# Seed shattering in common buckwheat (*Fagopyrum esculentum*): insights from RNA-seq and morphological analysis

**DOI:** 10.1186/s12870-025-07310-2

**Published:** 2025-10-14

**Authors:** Elizaveta M. Gunko, Mikhail I. Schelkunov, Sofia R. Prokopchuk, Anna V. Klepikova, Denis O. Omelchenko, Olga I. Romanova, Aleksey N. Fesenko, Ivan N. Fesenko, Aleksey A. Penin, Maria D. Logacheva

**Affiliations:** 1 Skolkovo Institute of Science and Technology, Moscow, Russia; 2 Vavilov Institute of General Genetics Russian Academy of Science, Moscow, Russia; 3 Federal Research Center N. I. Vavilov All-Russian Institute of Plant Genetic Resources (VIR), Saint-Petersburg, Russia; 4 All-Russian Research Institute of Legumes and Groat Crops, Orel, Russia

## Abstract

**Background:**

Seed shattering is a crucial adaptive trait in wild plant species but is undesirable in domesticated crops. In this study, we investigated the anatomical and molecular mechanisms underlying seed shattering in common buckwheat (Fagopyrum esculentum) and explored its genetic control. Previous research has shown that seed shattering in *F. esculentum* follows a monogenic inheritance pattern. To pinpoint the genomic region associated with this trait, we analyzed an F₂ progeny derived from a cross between wild ancestor of common buckwheat - *Fagopyrum esculentum* ssp. *ancestrale* and the cultivated variety - *Fagopyrum esculentum* cv. Dasha.

**Results:**

Anatomical analysis revealed that both shattering and non-shattering phenotypes possess an abscission zone (AZ); however, in non-shattering plants, the AZ remains underdeveloped and inactive. Histological observations indicated that AZ formation begins during early bud development. Transcriptomic analysis across four developmental stages (early bud, late bud, flower, and fruit) identified abscisic acid regulation, ethylene signaling, and cell wall modification as key factors in AZ differentiation. Unexpectedly, many genes typically associated with abscission layer activation showed increased expression at the flower stage rather than the fruit stage, suggesting that AZ activation occurs earlier than anticipated. We also provided preliminary data on the localization of the gene responsible for shattering in wild buckwheat and reviewed candidate genes.

**Conclusion:**

Our results provide new insights into the structure of abscission zone in buckwheat and into molecular mechanisms underlying seed shattering in buckwheat. We identified key pathways involved in abscission zone differentiation and performed the initial localization of the causative gene.

**Supplementary Information:**

The online version contains supplementary material available at 10.1186/s12870-025-07310-2.

## Introduction

Seed shattering is an important mechanism serving to disperse seeds far from the mother plant; it helps to reduce competition between plants of the same generation and to increase the general fitness. In domesticated cultivars however this trait is unfavorable because it leads to the loss of yield. As well as many other agriculturally important traits, it underwent selection independently in different species, being the part of the domestication syndrome. Cultivated cereals (rice, wheat, barley) are non-shattering, this trait is also valuable for breeding of tomatoes and legumes [1–3]. The shattering trait is realized due to the development of a specific structure called abscission zone (AZ). AZ is a group of specialized cells that are usually morphologically different from the neighboring tissues. They have a smaller size and dense cytoplasm, lack a secondary cell wall and have highly branched plasmodesmata [4–7]. In many cases, the abscission zone can be noticed with the naked eye as a narrowed ring between two organs.

Frequently, AZ cells themselves possess thin and not lignified cell walls, while the neighboring cells are thickly lignified. Thin and lignin-free cell walls are easier to degrade, and differential lignification creates a physical tension between cell types. When the cells of the abscission layer lose turgor pressure, this tension leads to the breakage in the weak layer. However, the lignification pattern is not the same for all species, and it is likely that there are different mechanisms for organ abscission [7–9].

The genetic basis of seed shattering is complex and non-conserved in different plant lineages, even within a family. Moreover, within one species several loci can affect shattering. In rice, the major gene conferring non-shattering is SH4. The SH4 locus is located on the fourth chromosome; it encodes a transcription factor with a Myb3 DNA-binding domain. In non-shattering cultivated rice the abscission layer forms incompletely because of mutation in SH4 . Most cultivars of rice, both from *indica* and *japonica* groups, carry this mutation. However, a weedy rice – a non-cultivated shattering variety derived from cultivated rice via dedomestication – also has the same mutation [10]. This shows that other genes can modify the action of SH4. Other genes that contribute to the shattering trait are SHAT1 [11] and qSH1 (SH5) [12]. Since rice is both an important agricultural plant and a model object, the downstream mechanisms underlying the action of these genes are extensively studied [13].

However, in other groups of grasses causative genes are not orthologous to these ones. In barley two linked loci, BTR1 and BTR2 , are the major determinants of shattering. The homozygous recessive genotype at any of the loci confers the non-shattering phenotype. Both are used in breeding: the majority of western barley cultivars have a recessive btr1 allele and dominant Btr2, while the most part of eastern cultivars have dominant Btr1 in combination with recessive btr2 allele. In emmer wheat, the non-shattering accessions carry mutations in Btr1 ortholog [14].

Much less is known about the genetic control of shattering in dicots. The most well studied object is tomato where several genes whose mutations confer non-shattering phenotypes are known. The first one discovered is JOINTLESS (J), a MADS-box gene with high similarity to regulator of flowering time SVP [15]. The *j* mutants do not develop an abscission zone; however, they also have changes in the inflorescence structure that are unfavorable for breeding. More recently another MADS-box gene, homolog of *Arabidopsis thaliana* SEP4, was found to be involved in shattering. The disruption of this gene, JOINTLESS2 (J2) confers the absence of AZ without adverse effects on inflorescence structure. Thus the loss-of-function allele of J2 is very widespread in cultivated tomato [2].

Besides shattering, which implies the detachment of the whole fruit, many plants have an alternative mechanism of seed dispersal – dehiscence – the opening of mature fruits and release of the seeds. This is the case for *A. thaliana*, where the silique, composed of two fused carpels, is opened at maturity in the dehiscence zone (DZ) which is formed at the fusion of the carpels. Shattering and dehiscence have a lot in common as they both rely on the formation of a zone with specific cellular characteristics, including the modification of cell walls. Two major actors – MADS-box genes SHATTERPROOF1 and SHATTERPROOF2 were identified [16]. Their homologs in *Brassica* crops were found to be responsible for pod shattering (see e.g. [17, 18]), emphasizing the common mechanisms underlying these processes.

Buckwheat, the object in this study, is a eudicot species from the order Caryophyllales, is an important grain crop, extensively grown in many Asian and European countries. Though it has lower yield than cereals, its grains have higher nutritional characteristics, being rich in proteins and many other valuable compounds and free of gluten (see e.g. [19]). Virtually all cultivated varieties of buckwheat are non-shattering; shattering is retained only in a wild variety, *F. esculentum* ssp. *ancestrale*. Despite the importance of this trait, very little is known about it, not only at the level of the genetic control but also on the level of the morphological and anatomical characteristics of the AZ. Fesenko and co-workers (1998) reported that seed shattering in *Fagopyrum esculentum* is controlled by a single gene and shattering is the dominant trait. This conclusion was based on the study of the F_2_ population derived from the cross between cultivated buckwheat and *F. esculentum* ssp. *ancestrale*, which gave a segregation of 3(shattering):1(non-shattering) [20]. Wang and co-workers (2005) draw a more complex picture with three genes involved [21]. This scheme, however, is based on a different material - interspecific hybrids between *Fagopyrum homotropicum* (wild shattering) and *F. esculentum* (cultivated, non-shattering) and may reflect the differences in the genetic background between these two species. At the level of morphology and anatomy, there is a single study specifically dealing with AZ, but the object of this study is non-shattering cultivar [22]. Thus, there is a significant gap in knowledge concerning anatomical structure of AZ in buckwheat as well as molecular determinants of the abscission process in this plant. The current study is aimed at filling this gap.

## Methods

### Anatomy

#### Plant material

Material from two accessions was collected in this study: cultivated *F. esculentum* cv. Dasha and wild subspecies *F. esculentum* ssp. *ancestrale*. Cultivated accession is non-shattering, wild accession is shattering. The specimens were grown in a greenhouse. Freshly harvested material was fixated in 75% ethanol. We collected the entire inflorescences and separated the flowers in lab conditions registering the phase of flowering. We selected three phases: a flower bud, an opened flower and a fruit. During flower separation we made a cut in close proximity to the main axis of inflorescence, taking the pedicel into further analysis. In case of big flowers and fruits we got rid of the biggest part of the reproductive organs to make the specimen more suitable for further manipulations.

#### Histological sectioning

Standard procedures were used for histological experiments [23]. See Table S1.

The staining proceeds directly without removal of the paraffin as follows:

1. The slides were placed in 1% aqueous safranin for 20 min.
2. The slides were rinsed in tap water to remove excess dye and rinsed for 1 min in distilled water. Excess water was blotted away.

3. The sections were counterstained in 0.5% fast green in 95% alcohol for 20–30 min.
4. The slides were thoroughly rinsed in tap water to remove excess dye and rinsed twice in distilled water for 1 min.

5. The excess water was blotted away and the slides were dried in a 37 °C oven for at least 30 min.
6. Paraffin was removed from the sections in two changes of limonene, each of 5 min, and the sections were mounted with a permanent resin.

#### Express test for lignin

To test the pedicels for the presence of lignin, we performed a phloroglucinol reaction using the classical protocol [24]. For staining we used the ethanol-preserved material. The visualization was performed using microscope Levenhuk MED PRO 600 Fluo for histological sections and Zeiss Stemi 2000-C microscope for morphology detalization.

### Transcriptomics

#### Plant material

The plants used for transcriptome analysis were obtained by hybridization of *Fagopyrum esculentum ssp. ancestrale* and *Fagopyrum esculentum* Dasha cultivar (F_3_ progeny). RNA was extracted from pedicels. For each phenotype (shattering/non-shattering) we analysed two biological replicates. Each replicate represented a pool of pedicels collected from at least five plants of the same phenotype; the number of pedicels within a pool was within the range of 20–30. We analyzed the transcriptome on four different stages of flower development. The stages 1–3 were collected from the inflorescences at the anthesis of the first flower. The stage 3 corresponded to the open flower (in buckwheat, flowers stay opened for one day). The stage 2 corresponded to the pedicel of a bud of the second flower in the inflorescence (the next after the one which is opened), the stage 1 - pedicel of a bud of the fourth flower. The stage 4 corresponded to the developing fruit when it becomes visible from the perianth but not yet began to turn brown.

#### Sequencing data

RNA libraries were prepared using NEBNext Ultra II sample preparation kit (New England Biolabs, USA) and sequenced on HiSeq4000 (Illumina, USA) platform. The length of the read is 150 bp from each end of the fragment with the sequencing depth 15-20 million reads per sample.

#### Gene expression analysis

The reads were mapped to the reference genome of *F. esculentum* ssp. KK8. KK8 is a self-pollinated accession of buckwheat for which we sequenced and assembled the genome (detailed genome analysis will be published elsewhere). The quality of assembly and annotation exceeds currently published buckwheat genomes [25–27], see Table S2. Read counts were calculated using STAR version 2.7.11b [28]. Normalization of read counts and differential expression analysis were done using DeSeq2 1.36.0 software [29]. The Gene Ontology enrichment of the DEGs was performed with GOAtools1.4.12 [30].

### Bulk segregant analysis

#### Search for association of shattering trait to genomic region

We used an F_2_ population derived from a cross between *Fagopyrum esculentum* ssp. *ancestrale* (wild ancestor) and *Fagopyrum esculentum* Dasha (a cultivated variety) which is segregating by the shattering trait. To develop this population, F1 was produced by crossing one cultivar Dasha individual to one *Fagopyrum esculentum* ssp. *ancestrale* individual using hand pollination. Then seeds (F1) were collected from parental plants, they were grown and subjected to random mating by insect pollination in order to produce F2. F3 used in transcriptomics analysis was produced out from this F2 population in a similar way as F2. The sample size comprises 206 non-shattering plants and 287 shattering plants, for a total of 493 plants. DNA extraction of F2 plants and of parental plants was performed using DiamondDNA Plant kit (DiamondDNA, Russia). Libraries were prepared using NEBNext Ultra II DNA for Illumina kit (New England Biolabs, USA). For sequencing we used HiSeq2000 or HiSeq4000 instrument (Illumina. USA) in 100 + 100 or 150 + 150 run mode. Each plant in the population was sequenced individually at low coverage, and the pools were formed computationally, by concatenating the raw reads of the corresponding phenotypic groups. Parental plants were sequenced with higher coverage, ~ 20x, in order to enable SNP calling, as well as several F_2_ plants.

Mapping was performed using BWA-MEM2 version 2.2.1, then we performed SNP calling using bcftools version 1.18 [31, 32]. The downstream analysis of SNP frequency changes across the genome was done using Qtl-seq version 2.2.4 [33]. To improve the results we developed a SNP filtering pipeline based on the consideration that parental lines are homozygous for the causative locus: *F. esculentum* ssp. *ancestrale* is homozygous by the allele that confers shattering and Dasha cultivar – for the allele conferring non-shattering. Using custom script (the script is available by the link: 10.5281/zenodo.15036179) we filtered the SNPs, retaining only those where the parents are homozygous and differ from each other.

#### Recombination check

For search of recombination events, we used VCF files of F_2_ plants sequenced with high coverage, retaining only the variants which were present and homozygous in either parent. Recombination events are detected by comparing adjacent genomic positions. Genotypes are initially represented in a format such as 0/0, 0/1, and 1/1, which correspond to homozygous reference, heterozygous, and homozygous alternative states, respectively. These genotypes are converted to numerical values (−1, 0, 1) for easier mathematical manipulation. Then we calculated the rolling median with the window size 100 bp, to get rid of the false SNPs. The table with median values was used in further steps. We identified the switches — points where the genotype transitions between different states. Possible transitions are [1 ->0], [−1 ->0], [0 ->1], [0 ->1]. Such transitions serve as markers of potential crossover events, where homologous recombination has reshuffled genetic material between parental chromosomes. To generate an overview of recombination activity across the genome we applied the algorithm to all descendant samples and chromosomes.

Working with this data, we also plotted the genotype itself, in order to see which genotype characterizes the shattering and non-shattering samples. The script for both described steps is provided by the link: 10.5281/zenodo.15036165.

#### Search for MADS-box genes

The MADS-box domain alignments were obtained from the InterPro database [34]. Genome-wide searches were conducted utilizing HMMer version 3.1b2 [35]. For these analyses, we used the annotated genome version encoded in amino acids: this approach inherently relies on the accuracy of existing genome annotations, which may contain inaccuracies. To complement this method, we used Miniprot-0.13 [36], which operates directly on genome assemblies. We retrieved a comprehensive list of *A*. *thaliana* MADS-box genes from the PANTHER database [37] and utilized their protein sequences to identify orthologous regions within the buckwheat genome. This approach yielded a set of genomic regions that likely represent orthologs of *A. thaliana* MADS-box genes.

The result of Miniprot usage was a list of genomic regions that are supposed to represent orthologs of *A. thaliana* MADS-box genes. The result of HMMer usage was a list of buckwheat gene identifiers associated with MADS-domain. We compared the genomic coordinates to match the outputs of two programs.

## Results

### Anatomy

**Fig. 1:**
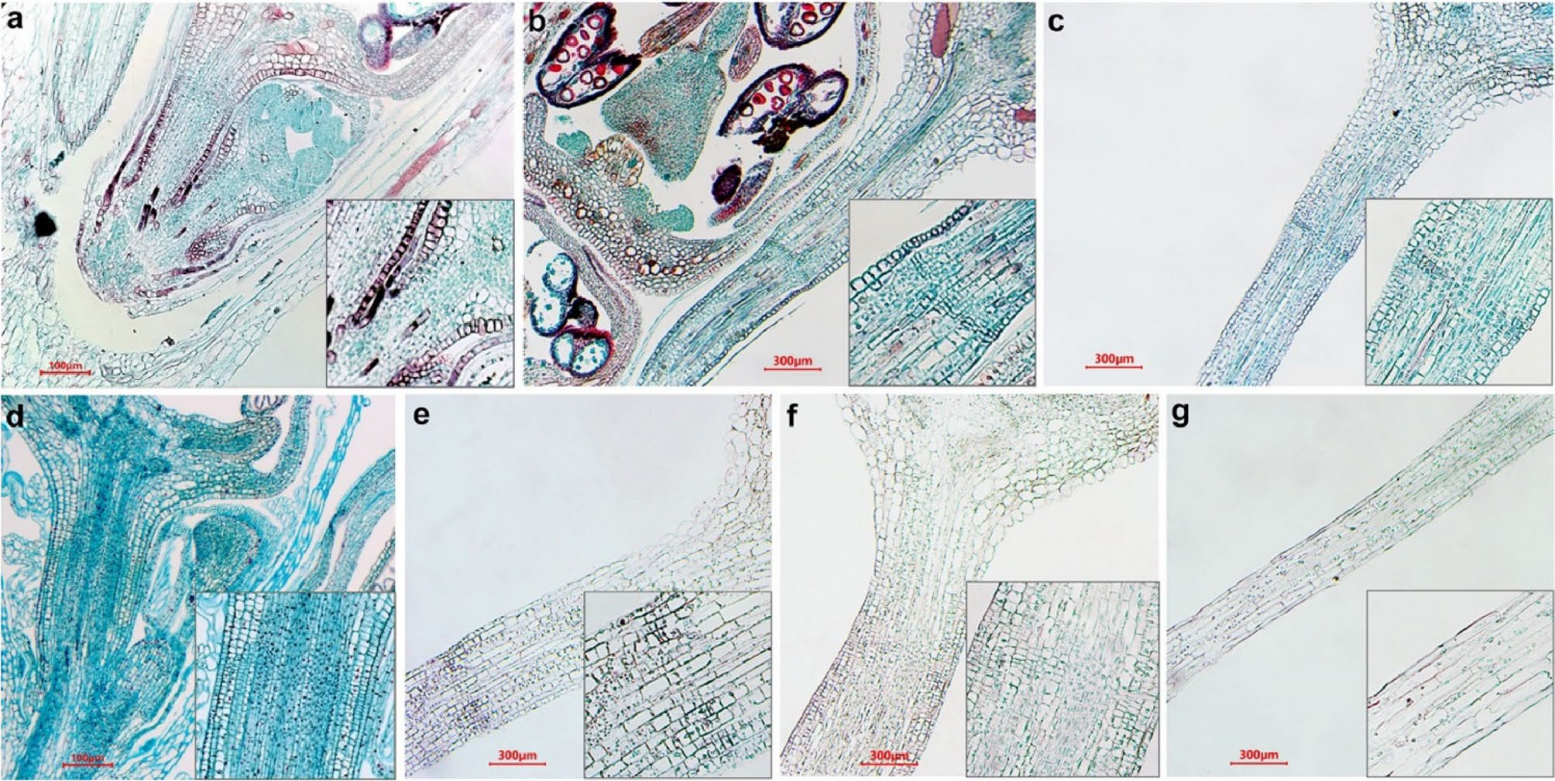
Comparison of *F. esculentum* ssp. ancestrale AZ with *F. esculentum* Dasha. Small window on each picture shows a bigger magnification of the AZ. **a*** F. esculentum* ssp. ancestrale early flower buds. **b*** F. esculentum* ssp. *ancestrale* late flower buds. **c*** F. esculentum* ssp. *ancestrale* mature flowers. **d*** F. esculentum* cv. Dasha early flower buds. This section was stained using alcian blue dye. **e*** F. esculentum* cv. Dasha late flower buds. **f*** F. esculentum* cv. Dasha mature flowers stage. **g*** F. esculentum* cv. Dasha pedicels on fruit stage

**Fig. 2:**
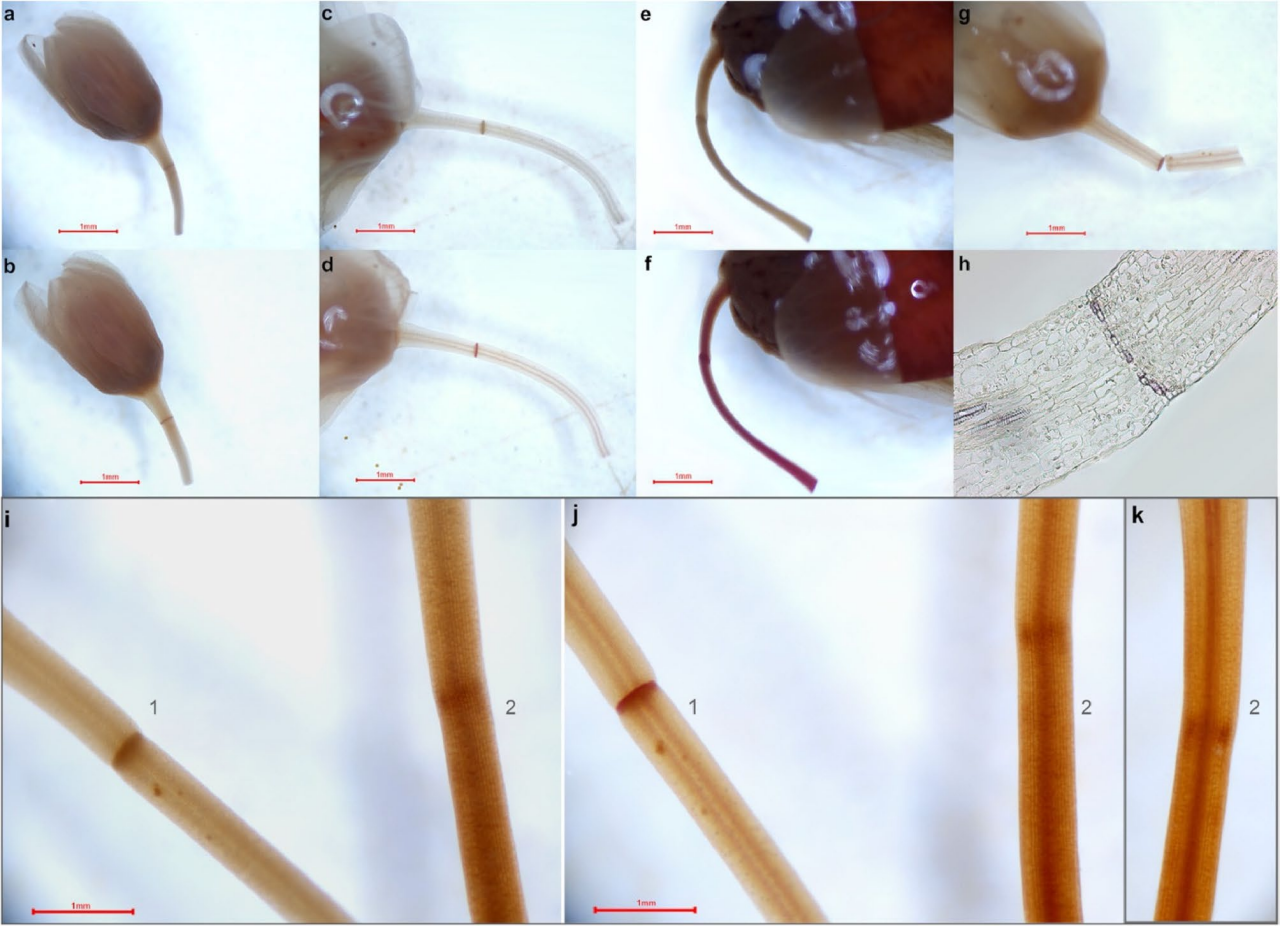
*Fagopyrum esculentum* lignin visualization. **a** - **h** - *F. esculentum* ssp. *ancestrale* (**a** - **b** flower bud, **c** - **d** mature flower, **e**-**f** fruit. **g** shows the AZ breaking during the flower stage, with the lignin layer positioned distally and lost together with the flower. **h** shows the single lignified layer in the flower pedicel. Pictures **a**, **c**, **e** are captured without staining, while pictures **b**, **d**, **f**, **g**, **h** demonstrate the result of phloroglucinol reaction. **i** – **k** - comparison of lignification patterns in *F. esculentum* ssp. *ancestrale* AZ and *F. esculentum* cv. Dasha. 1 – *F. esculentum* ssp. ancestrale, 2 – *F. esculentum* cv. Dasha. To enhance the staining effect, a longitudinal cut was made in F. esculentum cv. Dasha, as its thick pedicels hinder the reaction process. **i** Unstained pedicels. **j** Phloroglucinol-stained pedicels. **k** *F. esculentum* cv. Dasha viewed from the opposite side of the cut.

We examined two accessions of buckwheat: cultivated *F. esculentum* cv. Dasha and *F. esculentum* ssp. *ancestrale*. Unlike the cultivated buckwheat, F. *esculentum* ssp. *ancestrale* exhibits seed shattering. Comparing these accessions helps to understand abscission loss in cultivated *F. esculentum*. At the stage of the youngest flower buds, no distinguishable AZ is present in either *F. esculentum* ssp. *ancestrale* or *F. esculentum* cv. Dasha (Fig. 1a, d). Later in development the differences begin to emerge. In *F. esculentum* ssp. *ancestrale* there is a typical AZ, consisting of 4–8 layers of small cells **(**Fig. 1b, c, Fig. 2 a-h**)**. Lignin staining with phloroglucinol indicated a lignified layer on the distal side of the pedicels, closer to the flower **(**Fig. 2g-h**).**

In non-shattering *F. esculentum*, we noticed a structure morphologically resembling AZ. The presence of this structure is however, a variable trait; within a single inflorescence, some flowers have a distinct AZ, while others do not (Fig. S1). We analyzed the visibility of AZ depending on the flowering stage, using 236 flowers. Post-anthetic flowers have a visible AZ in 100% of cases, anthetic flowers have a visible AZ in 48.3% of cases and flower buds – in 95% of cases (Fig **S2****)**. However, even in case of most pronounced AZ in non-shattering samples lignification of AZ in their pedicels is absent (Fig. 2i-k, Fig. S1, S3).

When examining the histological sections of non-shattering samples, we found a structure morphologically resembling AZ only in flower buds and mature flowers **(**Fig. 1e, f**)**. But we didn’t observe anything similar in histological sections on the fruit stage **(**Fig. 1g**)**.

#### Transcriptome analysis

In total, we analyzed the transcriptome profiles of 16 samples, obtaining an average of 16.6 million paired reads per sample. The raw reads were trimmed and mapped to the reference genome. The statistics of trimming, mapping and counting of successfully assigned reads are listed in Table S3 and read counts – in Table S4.

**Fig. 3 Differential gene expression analysis:**
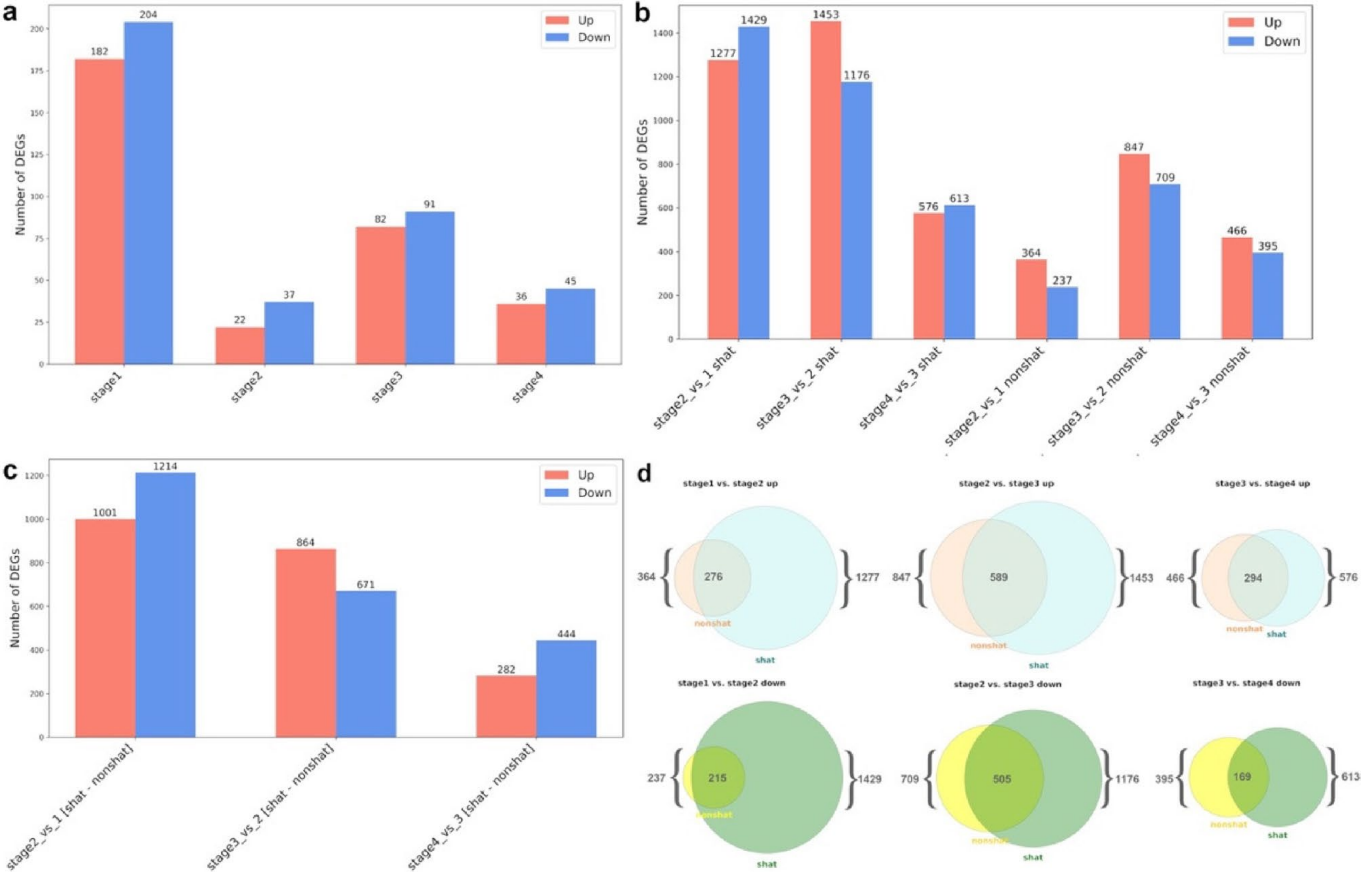
**a** The number of genes which are differentially expressed in shattering and non-shattering samples at each developmental stage. **b** DEGs across different stages within the same phenotype (shattering or non-shattering). The early stages of development show a higher number of DEGs compared to later stages, indicating significant transcriptional changes early in the development process. **с** DEGs unique for shattering samples in stages comparison. **d** Intersection of gene lists in shattering and non-shattering plants’ between stages comparison

Clustering of samples based on their expression profiles primarily reflects differences between developmental stages rather than phenotypes. Specifically, all samples formed two major clusters: one comprising samples from stages 1 and 2, and the other consisting of samples from stages 3 and 4 (Fig S4). We conducted a differential gene expression analysis between shattering and non-shattering samples, with non-shattering samples serving as the control and shattering samples as the experimental group. Additionally, we examined the temporal dynamics of gene expression in both sample types. As expected, the number of differentially expressed genes between shattering and non-shattering samples was low (Fig. 3a, for full list of DE genes see Tables S5-8), likely due to the close genetic relatedness of the plants within pools.

The largest difference between shattering and non-shattering samples was observed at the earliest stage of flower development. At the late bud stage, the number of differentially expressed genes (DEGs) significantly decreases. The flower stage shows a minor peak in DEG number, which is less pronounced than the peak observed during the early bud stage (Fig. 3a).

When comparing gene expression across developmental stages, shattering samples exhibit a higher number of DEGs at all stages compared to non-shattering samples. This is expected, as shattering samples not only progress from bud to flower and from flower to fruit but also develop an abscission zone (AZ). The most pronounced differences between phenotypic groups occur at the early and late bud stages. Shattering samples exhibit a high number of DEGs between stages 1 and 2, whereas non-shattering samples undergo relatively minor transcriptomic changes during this transition. Similarly, the “bud-to-flower” transition is marked by a significantly higher number of DEGs in shattering samples compared to non-shattering ones. This indicates that shattering samples experience more extensive transcriptional reprogramming during the early stages of flower development than non-shattering samples (Fig. 3b).

To better understand the dynamics of the development of the AZ we examined genes that were differentially expressed between stages exclusively in shattering samples (shattering-specific DEGs, for full list of DEG see Tables S9-11). These DEGs are expected to reflect transcriptional changes occurring specifically in shattering pedicels over time, independent of the overall flower ontogeny (Fig. 3c). The relative number of DEGs suggests that the most pronounced differences occur between stages 1 and 2, the bud-to-flower transition is less influenced by shattering-related processes. The flower-to-fruit transition provides the least information regarding abscission (Fig. 3c).

Non-shattering samples contained fewer “unique non-shattering genes” than shattering samples - “unique shattering genes”. Interestingly, the proportion of genes present in non-shattering samples but absent in shattering samples increases from bud to fruit stages (Fig. 3d). These observations suggest that, at early stages, non-shattering samples only transition from a small bud to a larger bud, whereas shattering samples undergo both bud growth and the formation of a functional abscission layer. Nearly all genes found in the non-shattering phenotype also appear in the shattering phenotype, indicating that only a small fraction of genes accounts for individual effects. By the later stages, the influence of the abscission process decreases, while the role of individual genetic effects becomes proportionally more pronounced (Fig. 3d).

In order to gain insights into the functional roles of DE genes and their involvement in shattering we performed Gene Ontology enrichment analysis. First we focused on the differentially expressed genes (DEGs) identified in comparisons between shattering and non-shattering plants at the same stage of development. Despite low number of DE genes, enriched GO terms were present at every stage. The number of enriched terms was lower in stages 3 and 4 (Table 1). The plots can be found in Fig. S5.

In early flower buds the most prominent is the overrepresentation of the genes which are involved in the light phase of photosynthesis and in lignin metabolism. In late buds there are clear signs of stress response: such as overrepresented “response to heat” and “protein folding chaperone” terms. Stress response is continuing on the flower stage, as it follows from the overrepresented “response to heat” term. We interpret this as a result of the interplay of temperature stress and abscission pathways (see discussion below). Also, flower stage is characterized by the deficiency of “xylan biosynthetic process” and “hemicellulose metabolism process” which likely points at the process of AZ activation and will be discussed later.

**Table 1:** Enriched GO terms, found among genes, differentially expressed in shattering samples comparing to non-shattering ones

	GO	NS	Name	Ratio in study	Ratio in background	p_bonferroni	p_fdr_bh	gene_ids
Stage1 Up	GO:0009765	BP	photosynthesis, light harvesting	5/118	18/17,618	0.000451	0.000451	g26092, g28152, g2827, g29406, g29407
	GO:0019748	BP	secondary metabolic process	13/118	440/17,618	0.0362	0.0181	g1029, g13629, g16399, g18680, g21531, g21936, g23558, g24053, g27593, g28693, g373, g5402, g9168
	GO:0046274	BP	lignin catabolic process	4/118	27/17,618	0.136	0.0453	g18680, g28693, g373, g9168
	GO:0009522	CC	photosystem I	5/118	24/17,618	0.000407	0.000407	g26092, g28152, g2827, g29406, g29407
	GO:0009523	CC	photosystem II	5/118	43/17,618	0.00832	0.00416	g26092, g28152, g2827, g29406, g29407
	GO:0009521	CC	photosystem	5/118	51/17,618	0.0195	0.00649	g26092, g28152, g2827, g29406, g29407
	GO:0046906	MF	tetrapyrrole binding	15/118	497/17,618	0.00301	0.00301	g11291, g13354, g14608, g17699, g26092, g27741, g28152, g2827, g28725, g29406, g29407, g29482, g5402, g6346, g7720
	GO:0016168	MF	chlorophyll binding	5/118	33/17,618	0.00656	0.00328	g26092, g28152, g2827, g29406, g29407
	GO:0052716	MF	hydroquinone: oxygen oxidoreductase activity	4/118	27/17,618	0.0772	0.0257	g18680, g28693, g373, g9168
Stage1 Down	GO:0031464	CC	Cul4A-RING E3 ubiquitin ligase complex	3/141	11/17,618	0.0676	0.0225	g11770, g23160, g7420
	GO:0009514	CC	glyoxysome	2/141	3/17,618	0.162	0.0406	g10421, g26171
	GO:0043232	CC	intracellular non-membrane-bounded organelle	0/141	1210/17,618	0.0629	0.0225	g10416, g11251, g11303, g11312, g11330, g11337, g12236, g12424, g15458, g1547, g15756, g15800, g17023, g17039, g17208, g17637, g19109, g19274, g20348, g21265, g21465, g21756, g22283, g2231, g22362, g22884, g23599, g24380, g24414, g24650, g25350, g25783, g26163, g26354, g27131, g27184, g28192, g28193, g28194, g28720, g29147, g29512, g3408, g3577, g410, g4376, g5049, g5608, g5900, g6688, g6874, g7172, g9234, g9235, g9563, g9604, g9738
	GO:0043228	CC	non-membrane-bounded organelle	0/141	1230/17,618	0.0652	0.0225	g10716, g14691, g14706, g15008, g15079, g15510, g18340, g18697, g19436, g20241, g20936, g22316, g24080, g24622, g26301, g26788, g28525, g28799, g5418, g590
	GO:0005506	MF	iron ion binding	13/141	383/17,618	0.0329	0.0275	g12676, g13015, g15435, g15764, g16642, g17592, g22059, g23516, g2358, g23910, g2535, g27831, g7181
	GO:0020037	MF	heme binding	14/141	463/17,618	0.055	0.0275	g12676, g1272, g13015, g15435, g15764, g16642, g21558, g22059, g2358, g23910, g2535, g27831, g5020, g7181
	GO:0046906	MF	tetrapyrrole binding	14/141	497/17,618	0.119	0.0335	g12676, g1272, g13015, g15435, g15764, g16642, g21558, g22059, g2358, g23910, g2535, g27831, g5020, g7181
	GO:0004497	MF	monooxygenase activity	12/141	377/17,618	0.134	0.0335	g12676, g13015, g15435, g15764, g16642, g22059, g2358, g23910, g2535, g25385, g27831, g7181
	GO:0033612	MF	receptor serine/threonine kinase binding	5/141	54/17,618	0.183	0.0341	g12531, g12882, g3587, g7910, g8081
	GO:0001653	MF	peptide receptor activity	3/141	11/17,618	0.205	0.0341	g12531, g3587, g7910
Stage2 Up	GO:0006457	BP	protein folding	5/17	215/17,618	0.00647	0.00647	g11465, g12504, g22555, g25567, g27125
	GO:0051259	BP	protein complex oligomerization	3/17	40/17,618	0.0329	0.012	g11465, g12504, g15399
	GO:0009408	BP	response to heat	4/17	138/17,618	0.0361	0.012	g11465, g12504, g25567, g27125
	GO:0009266	BP	response to temperature stimulus	5/17	342/17,618	0.0624	0.0156	g11465, g12504, g22555, g25567, g27125
	GO:0051082	MF	unfolded protein binding	4/17	105/17,618	0.00693	0.00693	g11465, g12504, g25567, g27125
	GO:0140662	MF	ATP-dependent protein folding chaperone	3/17	60/17,618	0.064	0.032	g22555, g25567, g27125
	GO:0044183	MF	protein folding chaperone	3/17	75/17,618	0.125	0.0417	g22555, g25567, g27125
Stage2 down	No enriched GO terms were found							
Stage3 Up	GO:0009408	BP	response to heat	6/60	138/17,618	0.0334	0.0334	g12802, g13826, g14322, g21194, g28633, g28751
Stage3 Down	GO:0045491	BP	xylan metabolic process	6/69	102/17,618	0.0132	0.0132	g16197, g19833, g21092, g26940, g361, g5758
	GO:0045492	BP	xylan biosynthetic process	4/69	39/17,618	0.0729	0.0364	g19833, g26940, g361, g5758
	GO:0010410	BP	hemicellulose metabolic process	6/69	148/17,618	0.112	0.0374	g16197, g19833, g21092, g26940, g361, g5758
Stage4 Up	No enriched GO terms were found							
Stage4 Down	GO:0009514	CC	glyoxysome	2/37	3/17,618	0.011	0.011	g10421, g26171

GO terms are filtered by the p_fdr_bh< 0.05. Stage1 – early buds, stage2 – late buds, stage3 – mature flowers, stage4 – fruits

**Fig. 4:**
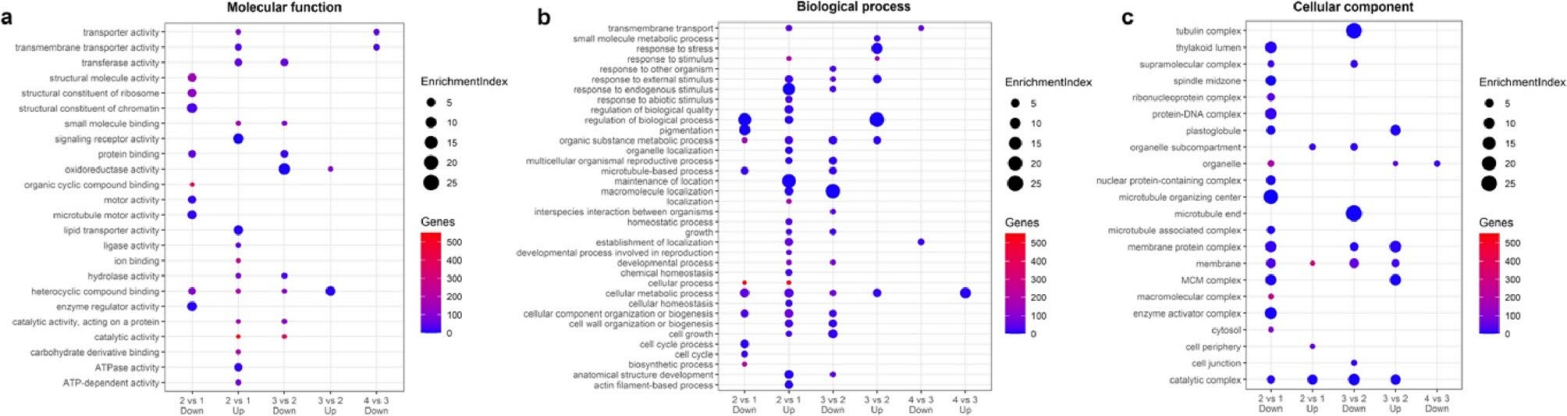
Enriched GO terms (third level only) present in shattering-specific genes. **a** Molecular function (MF). **b** Biological process (BP) **c** Cellular component (CC)

As part of a multi-step comparison aimed at the identification of genes unique to shattering samples, we first evaluated differential expression across multiple stages in both shattering and non-shattering samples, then excluded any genes also found to be differentially expressed in non-shattering (see above). We used this refined set of shattering-specific genes for GO enrichment analysis. The terms with the highest enrichment index (calculated as [Ratio in DE set/Ratio in all genes]) are shown in Fig. 4. The table including all enriched terms, with genes and their annotations is provided in Tables S12-S14.

In late buds compared with early buds, primary cell wall biogenesis is clearly activated; processes such as (1→3)-β-D-glucan biosynthesis and cutin transport are likely involved. Molecular function data also support these findings, pointing to (1→3)-β-D-glucan synthase and cellulose synthase activities. Unexpectedly, one of the most strongly enriched terms is “pollen tube development.” However, upon examining the annotations of individual genes contributing to this term, we found that they are homologs of genes encoding enzymes involved in cell wall component synthesis—for example, xyloglucan glycosyltransferases (g19065, g22558, g24241) — or in cell wall biogenesis more broadly, such as cellulose synthase-interacting proteins (g13513, g6320) and genes related to cuticular wax biosynthesis. None of these genes appear to be directly associated with pollen-specific processes. Thus, we infer that this enrichment is likely an artifact arising because these proteins are indeed active during pollen development in model species, where extensive cell wall reorganization occurs. However, this function may not be conserved or exclusive to pollen development in buckwheat. Thus this overrepresentation rather reflects the processes of modification of cell walls during formation of AZ. In addition, there is evidence of ethylene response, as well as reinforced calcium signaling. Apart from that, cell division intensity decreases from early to late buds, this is reflected in the downregulation of genes related to cell replication, DNA biosynthesis, and cell-cycle phase transition, including cyclin-dependent protein kinases. We also observe a decline in photosynthesis-related processes in late buds. The enrichment of cellular component terms is highly congruent with findings described above. It shows overrepresentation of the term “trans-Golgi network” – this is a subcompartment which is key for the biosynthesis and trafficking of cell wall components. At the same time, the terms related to photosynthesis and cell division are overrepresented in downregulated DEG, emphasizing the suppression of photosynthesis and the prevalence of cell differentiation over cell division at this stage. In flowers compared with late buds, photosynthesis shows some activation again, as well as fatty acid catabolic processes. In contrast to earlier stage, the processes related to cell wall biogenesis, in particular, pectin and cellulose biosynthetic processes are downregulated. The same is evident in the cellular component terms: ones that are related to cell wall and Golgi apparatus are overrepresented within downregulated DEG. The fruit stage has the lowest number of shattering-unique genes; congruent with this, there is almost no overrepresented terms in either up or downregulated DEG. The only few exceptions are lignin metabolic process in upregulated genes and transmembrane transport related processes in downregulated genes (Fig. 4).

### Genetic analysis and gene mapping

Since previous data on the inheritance of shattering trait were contradicting, we first performed a phenotypic survey of F2 plants derived from a cross between *Fagopyrum esculentum ssp. ancestrale* and *Fagopyrum esculentum* cv. Dasha. We registered 77 non-shattering and 262 shattering plants which corresponds to monogenic inheritance with non-shattering being recessive (chi-square = 0.95, less than critical value at *p* = 0.05).

To identify the genomic region responsible for seed shattering, we then used a bulk-segregant analysis approach. This method involves whole-genome sequencing of pools of individuals from a segregating progeny that exhibit extreme opposite trait values for a given phenotype. We used a sampling of ~ 200 plants of each phenotype from F_2_ population for pooled sequencing and processing using QTL-seq pipeline [33].Also, we selected from the same population 28 plants with shattering phenotype and 4 plants with non-shattering phenotype for individual high-coverage sequencing.

**Fig. 5:**
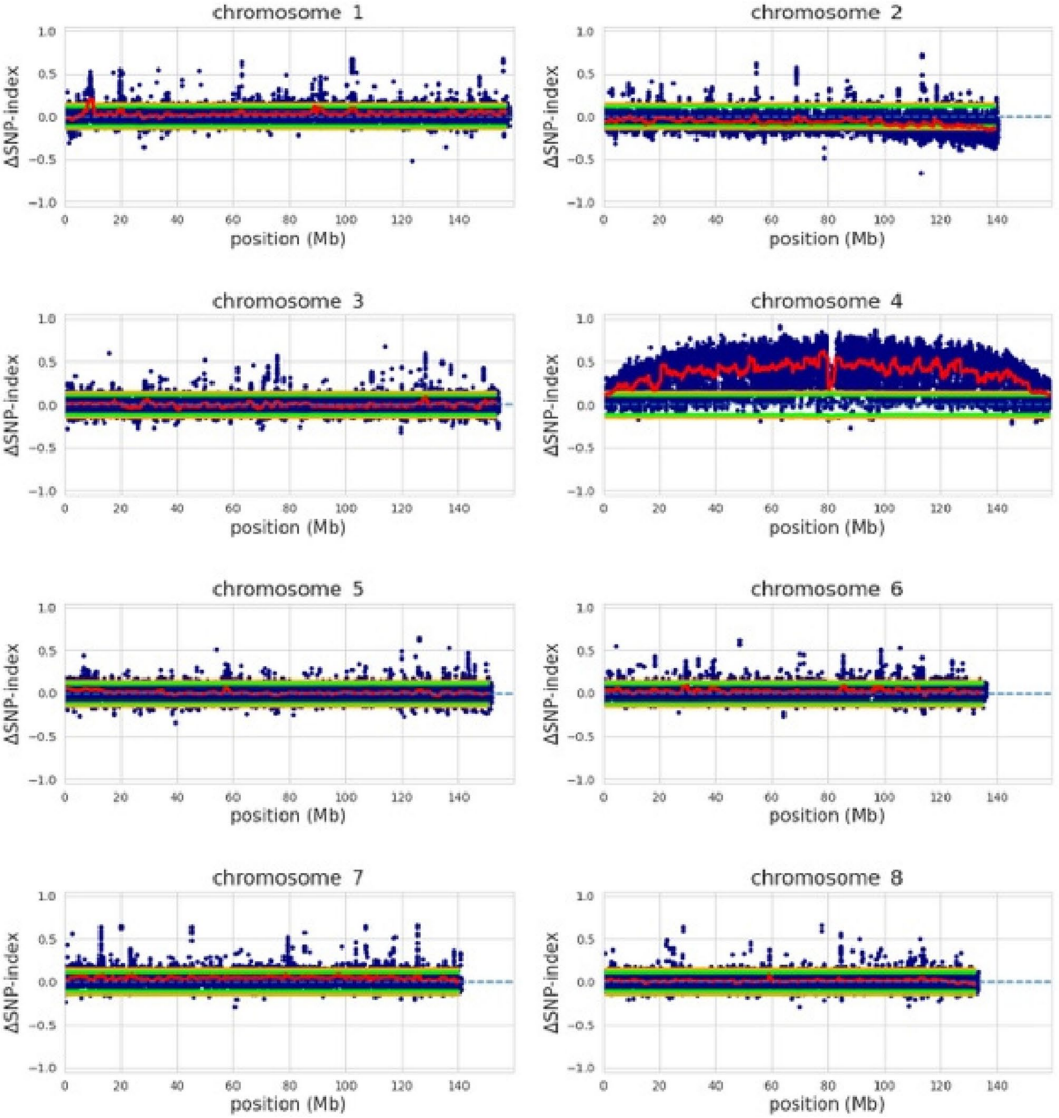
Results of QTL-seq. Single nucleotide polymorphism (SNP)-index plots of ∆SNP-index plot with statistical confidence intervals under the null hypothesis of no QTLs (red, mean SNP-index; green, *P* < 0.05; yellow, *P* < 0.01). This picture illustrates the specifically filtered SNPs, number of SNPs = 193,958

According to QTL-seq results, the causative gene is located on the 4th chromosome (see Table S2 to check for chromosome correspondence in other genome assemblies). After the filtration by quality and coverage, and the specific filtration procedure based on the genetic requirements for the trait of interest, the number of SNPs was reduced from 35,821,013 to 193,958, but the localization peak remained broad **(**Fig. 5, **Fig. S6).**

**Fig. 6:**
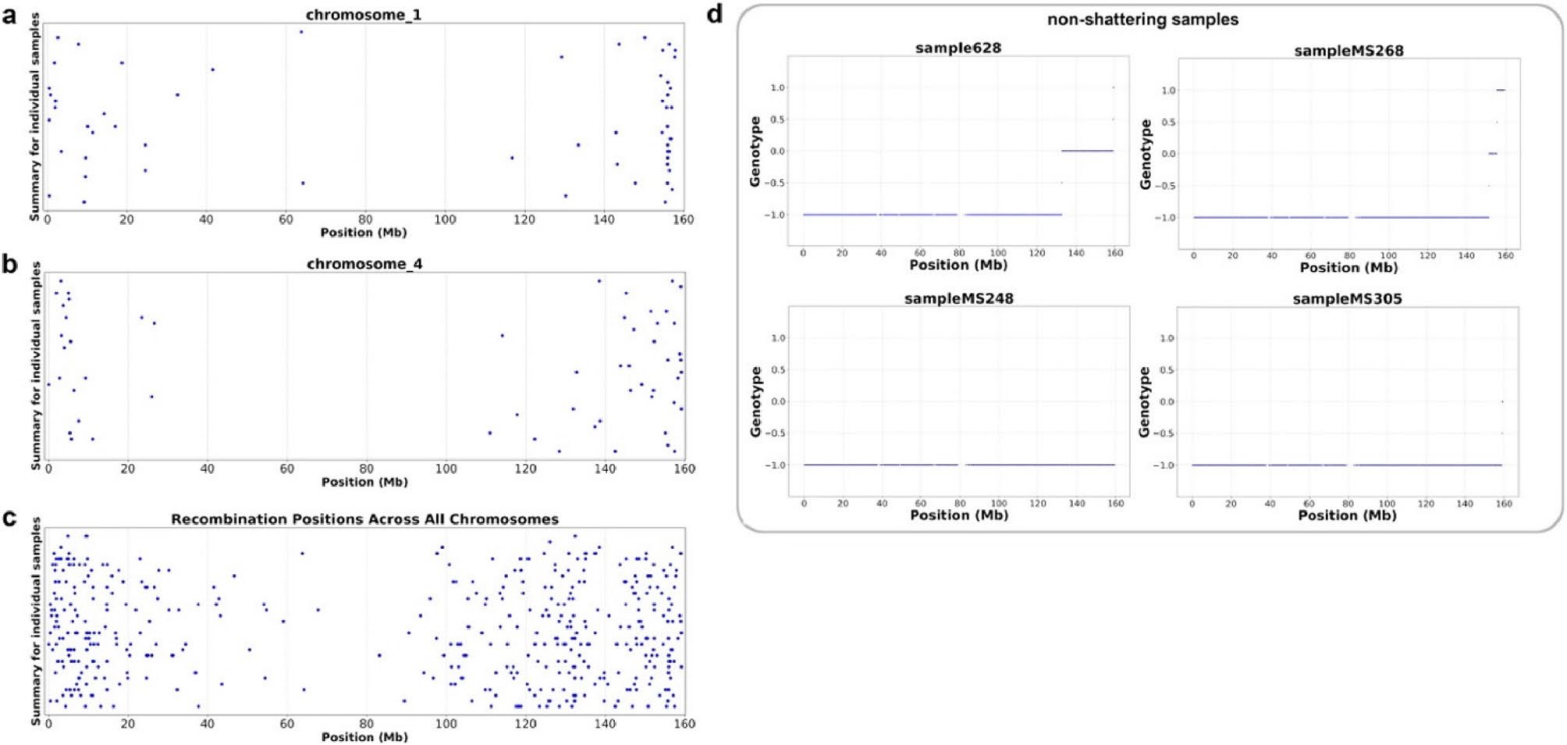
**a**,** b**,** c** diagrams illustrating the distribution of crossover locations across chromosomes. Each point represents a crossover event in one of the 32 individually sequenced F2 descendants (*Fagopyrum esculentum* ssp. *ancestrale* × *Fagopyrum esculentum* cv. Dasha). Y-axis is used to space out the samples. **a** Indicates crossover events on the 4th chromosome, **b** - on the 1st chromosome, **c** - summarizes recombination events in all eight chromosomes. **d** Genotype plots of non-shattering plants, Chr 4. The X-axis represents the position along the chromosome, the Y-axis shows the genotype: «1» indicates homozygous alleles differing from the reference, «0» indicates heterozygous alleles, and «−1» indicates homozygous alleles identical to the reference. Separate plots represent the non-shattering individuals. The reference genome is a non-shattering cultivar, and the observed genotype distribution aligns with expected inheritance patterns

One possible explanation for this pattern is a lack of recombination events on chromosome 4. To check this hypothesis, we used high coverage sequencing data from individual F_2_ descendants of the cross between *F*. *esculentum ssp. ancestrale* and *F. esculentum* cv. Dasha. Using a custom script, we calculated the number of recombination events on chromosome 4 and compared them with other chromosomes in the same samples. In total, we detected 403 crossovers across 32 samples, corresponding to an average of 1.6 crossovers per chromosome per generation (a file listing all detected crossovers is provided in **Table S15**). Our results indicate that crossovers occur more frequently at the ends of chromosomes **(**Fig. 6a-c**)** while the causative region is likely to be located closer to the middle of the chromosome, near the centromere (in buckwheat all chromosomes are metacentric or submetacentric).

Using high-coverage sequencing of individual plants, we analyzed shattering and non-shattering samples separately. Our objective was to identify crossover locations on chromosome 4 in the non-shattering samples. Among the four non-shattering samples, two inherited an intact chromosome 4 from Dasha, while the other two had crossovers. In the recombinant samples, crossovers occurred only at the ends of the chromosome **(**Fig. 6d**)**. For the shattering samples, some inherited the full chromosome 4 from the *F. esculentum* ssp. *ancestrale* parent, while others were recombinant. Similarly to the non-shattering samples, recombination in the shattering samples occurred at the chromosome ends (Fig. S7).

### MADS-box genes

Based on available data on other dicots, known regulators of shattering belong to MADS-box genes. To find candidate genes for the shattering locus we searched for MADS-box genes in buckwheat genome using HMMer and Miniprot [35, 36].

**Table 2:** MADS-box genes identified in chromosome 4 of *Fagopyrum esculentum* KK8 genome assembly. ^a^average expression stands for the mean amount of read counts across shattering samples in all stages of flower development (from buds to fruits), ^b^coordinates are given in descending order if the gene is located on minus strand

gene ID	Relevant info from functional annotation	Best blast hit	Average expression^a^	Coordinates^b^
g13053.t1	-	XP_021717252.1 agamous-like MADS-box protein AGL62 [Chenopodium quinoa]	12	34,302,816–34,303,400
g13071.t1	-	XP_021717252.1 agamous-like MADS-box protein AGL62 [Chenopodium quinoa]	1	34,836,036 − 34,835,452
g13074.t1	-	XP_021717252.1 agamous-like MADS-box protein AGL62 [Chenopodium quinoa]	11	35,103,266–35,103,853
g13446.t2	MADS transcription factor, positive regulation of transcription by RNA polymerase II, negative regulation of brassinosteroid mediated signaling pathway	XP_021662492.1 MADS-box protein SVP [Hevea brasiliensis]	262	63,152,951 − 63,150,847
g13503.t1	Transcription factor MADS-box, positive regulation of transcription by RNA polymerase II, regulation of pollen tube growth, pollen maturation	KAB1199747.1 MADS-box transcription factor 21 [Morella rubra]	0	72,634,616 − 72,633,014
g14066.t1	-	XP_031386029.1 MADS-box protein FLOWERING LOCUS C-like isoform X1 [Punica granatum]	51	126,038,449 − 126,023,023
g14190.t1	Agamous-like MADS-box protein AGL65 isoform X1, positive regulation of transcription by RNA polymerase II nucleus	KAG2702397.1 hypothetical protein I3760_06G087100 [Carya illinoinensis]	63	133,925,205–133,927,516
g14228.t2	-	AKI81897.1 APETALA1-like protein [Fagopyrum esculentum]	2387	135,393,121–135,394,679
g14614.t1	-	XP_021735939.1 MADS-box protein SOC1-like [Chenopodium quinoa]	110	147,355,797 − 147,351,163
g14773.t2	FRUITFULL-like MADS-box (Fragment), positive regulation of transcription by RNA polymerase II	AKI81899.1 FRUITFUL-like protein [Fagopyrum esculentum]	97	151,077,597 − 151,074,485
g15048.t1	-	XP_028788383.1 agamous-like MADS-box protein AGL62 [Prosopis alba]	0	155,283,097 − 155,282,333
g15049.t1	-	XP_041013322.1 agamous-like MADS-box protein AGL62 [Juglans microcarpa x Juglans regia]	0	155,298,835 − 155,298,071
g15050.t1	-	MBA0786926.1 hypothetical protein [Gossypium trilobum]	0	155,313,428 − 155,312,661
g15081.t1	MADS-box transcription factor 23-like isoform X2, RNA polymerase II transcription regulatory region sequence-specific DNA binding, positive regulation of transcription by RNA polymerase II	KNA23361.1 hypothetical protein SOVF_025490 [Spinacia oleracea]	0	155,796,304–155,796,485
g15082.t1	MADS-box transcription factor ANR, positive regulation of transcription by RNA polymerase II, cellular response to nitrate, lateral root development, response to nutrient	XP_021862467.1 MADS-box transcription factor 23-like isoform X1 [Spinacia oleracea]	0	155,804,940–155,809,898

The table containing full results is listed in Table S16. Overall, we found 84 MADS-box genes across buckwheat genome. This is within the range of the number of MADS-box genes in other flowering plants [38]. At the fourth chromosome whether the shattering locus is located, we found 15 MADS-box genes (Table S16). We hypothesize that the causative gene should be expressed in the pedicels so we examined their expression in our samples. Based on its localization within a 10–120 Mb region and the presence of a non-zero read count signal, we have identified four candidate genes: g13053, g13071, g13074, and g13446 **(**Table 2**).**

## Discussion

### Anatomy

An obvious hypothesis about the difference between shattering and non-shattering buckwheat accessions is the presence of an abscission zone (AZ): a plant with AZ exhibits a shattering phenotype, while a plant without AZ does not. However, as our experimental results show, this is not exactly the case. Instead, the shattering phenotype is determined by the presence of an active AZ, while non-shattering plants also possess AZ, but it is underdeveloped and inactive, with its level of expression varying among flowers even within the same inflorescence **(**Figs. 1d-g and 2, Fig. **S1**, S2). This is congruent with earlier observations by Fesenko NV [39] that Russian buckwheat cultivars have an AZ, but it is not functional. The study by Oba et al. 1998 that investigated several Russian, Japanese and Nepalese accessions failed to identify in the pedicels any specific structure that could be attributed to an AZ suggesting that there is a polymorphism between accessions [22]. The studies on other crops (in particular grasses) that have shattering and non-shattering accessions suggest that shattering is not always a binary trait, it is rather a spectrum where different degrees of shattering are based on different structures of AZ (see e.g. recent study on rice [40]). Considering the agricultural importance of this trait, it is of crucial importance to characterize its variation. In buckwheat the only accession that develops fully functional AZ is *F. esculentum* ssp. *ancestrale*. AZ appears in the early bud stage, before the flowering starts. The structure of AZ is quite similar to the ones, which are described for other species [5, 41]. The number of layers in the AZ is highly variable among species; *F. esculentum* has 4–8 layers of cells in the AZ **(**Fig. 1, b-c**)**. These cells are often referred to as meristem-like due to their small size and dense cytoplasm [4–7]. The lignification pattern is another feature that varies over species, sometimes it is absent [7, 42, 43]; in our case a lignified embedment is located in the distal part of pedicel, and abscission happens in such a way that the lignified layers are lost with the fruit **(**Fig. 2g**).**

In non-shattering buckwheat cultivar Dasha the AZ is present but not fully developed as evidenced by the histological sections. The structure observed in the non-shattering samples appears more like cell heterogeneity rather than a fully formed AZ. Also, it is not lignified. Binocular visualization further reveals inconsistent expression of the trait, with significant variation even within the same inflorescence. Apparently, inconsistency is associated with non-functionality – in *F. esculentum* ssp. *ancestrale* all investigated pedicels were monomorphic, with clearly visible lignified AZ.

The differentiation and activation of an AZ is a long process which includes many intermediate steps. Any kind of splitting the chain of events can lead to the occurrence of non-shattering phenotype. For instance, such rice mutants as *shattering abortion1* (*shat1)*, *shat2*/*sh4*, *sh5*, and *osh15* have reduced seed shattering due to the excessive lignification of the AZ [7]. Not only the excess of lignin, but the absence of lignin can also disable the abscission, as it happens in *A. thaliana shp1 shp2* double mutant and in *indehiscent* mutant [16, 44]. Other reasons can be the absence of the separation layer inside AZ - for example, *A. thaliana alcatraz* mutant, or the absence of the whole AZ - tomato *jointless* mutant [15, 45]. In some cases, the non-shattering phenotype can be caused by the mutations in genes coding for cell wall catabolic enzymes, which act in the latest stage of abscission - ARABIDOPSIS DEHISCENCE ZONE POLYGALACTURONASE1 (ADPG1) and ADPG2. *adpg1 adpg2* double mutants represent this kind of indehiscent plants [46]. In *F. esculentum* shattering is a monogenic trait (at least when inheritance is studied based on crosses with *F. esculentum ssp. ancestrale*), while in closely related species *F. tataricum* it is digenic [20, 47]. At the same time, non-shattering *F. tataricum* accessions do not have any AZ at all. We thus hypothesize that there are two major loci contributing to shattering – one controls the initial formation of the AZ and the second – its further differentiation and activation. In non-shattering *F. tataricum* both are mutated while in non-shattering *F. esculentum* cultivars – only one.

### Gene expression analysis

Transcriptome analysis proved to be useful in showing how the shattering and non-shattering plants differ at each stage of development and how the shattering phenotype unfolds through time. Two main groups of processes, inferred from gene expression changes, are evident in the results of gene expression analysis: ones related to hormonal response and to the cell wall.

At first stage, in early buds, gene ontology enrichment analysis revealed that the genes which account for light phase of photosynthesis and lignin metabolic process, are upregulated. The observed overexpression of Chl a/b binding proteins can imply for the presence of abscisic acid (ABA). The light-harvesting chlorophyll *a*/*b*-binding (LHCB) proteins are a part of antenna complex bound to photosystem II. It was shown that LHCB proteins are positively involved into ABA regulation. LHCB and ABA mutually enhance each other [48]. Thus, we suggest that the presence of Chl a/b binding proteins points on the beginning of ABA response, rather than intensified photosynthesis. Abscisic acid 8’-hydroxylase, a cytochrome P450 monooxygenase, is downregulated at this stage. This enzyme catalyzes the first step of ABA catabolism [49]. As it is evident even from the name, abscisic acid is a known regulator of abscission. However, the conception of ABA role in abscission processes has significantly changed during the last decades. The effect of ABA greatly depends on auxin and ethylene regulation. Ethylene appears to have a deciding role in activating the cell wall detachment, while auxin and ABA mostly function as as intermediate regulators in this process [4, 50, 51].

Following the transcriptional patterns suggestive of ABA-related events, the late buds stage in shattering samples showed upregulation of 9-cis-epoxycarotenoid dioxygenase (NCED) gene. The NCED protein catalyzes the key step in ABA biosynthesis. Interestingly, we didn’t observe it among up-regulated ones in the earliest stage. In 2021 Lang and the others investigated ABA involvement into abscission process in rice. In their study, ABA levels were nearly equal in shattering and non-shattering rice samples at the earliest stage of inflorescence development, but later, ABA levels rose more significantly in shattering samples. Transcriptomic analysis of the samples demonstrated that the expression of OsNCED coincides with the increased ABA content [52]. While we did not quantify ABA content in our study, our transcriptomic data on NCED upregulation in late buds could hypothetically align with increased ABA levels in shattering pedicels as well Heat shock proteins (HSPs) encoding genes are upregulated in late buds, flowers and fruits. Many studies show that HSPs are induced by a variety of environmental stimuli. Stresses never come alone, and heat stress is tightly linked with high light and draught. Some HSPs are known to be involved in developmental processes, seed germination and fruit maturation [53]. HSPs are often induced by ABA [54]. Based on the observed gene expression patterns, we speculate that the abundance of HSPs in these stages of shattering samples is related to the activation of an intensified ABA response.

Our transcriptomic data also indicate patterns consistent with ethylene and auxin hormonal interplay. In the comparisons between phenotypes within the same stage, the genes of auxin response factors are downregulated in buds and in flowers (Table S5, S6). On the other hand, ethylene response genes are upregulated in the late buds comparing to early buds in shattering samples (Fig. 4a). Auxin is transported from distal organs (flower or fruit) and it suppresses abscission [55, 56]. However, developmental factors lead to a decrease in basipetal auxin transport, making AZ cells more sensitive to ethylene [55, 57–59]. Wang and others (2021) describe the mechanism of action of a certain ethylene response factor - SlERF52 (ETHYLENE RESPONSE FACTOR 52), and in particular, its role in tomato fruit abscission. SlERF52 is a downstream target of transcription factors MACROCALYX (MC) and JOINTLESS (J), SlERF52 expression is suppressed in plants with disturbed MC and J function [60]. SlERF52 is specifically expressed in the pedicel AZ and plays an important role in regulating the abscission process. Its function is linked to the activation of cell wall-degrading enzymes, such as polygalacturonases (PGs) and cellulases (Cel), which are necessary for cell separation and also it affects meristematic gene expression, including genes like WUSCHEL homolog (LeWUS), GOBLET (GOB), and LATERAL SUPPRESSOR (Ls) [61]. The research of Wang et al. 2021 has further linked SlERF52 to aquaporin regulation, demonstrating that it directly controls the expression of SlTIP1;1, a tonoplast intrinsic protein crucial for abscission. This tonoplast intrinsic protein (TIP) is localized in both the plasma membrane and tonoplast, facilitating the movement of H_2_O_2_ into the cytoplasm. Elevated H_2_O_2_ concentrations suppress auxin signaling and enhance ethylene production, forming a positive feedback loop with ethylene. This shift helps the AZ cells to transition from an auxin-dominant stage to an ethylene-responsive stage. Also, SlTIP1;1 facilitates water influx into AZ cells, generating turgor pressure, which is needed for cell expansion and the mechanical force required for organ detachment [59]. Tonoplast intrinsic protein is present among downregulated shattering-specific genes in comparison between flowers vs. late buds (g21653) and fruits vs. flowers (g22543, g10638, also see GO enrichment terms like “inorganic molecular entity transmembrane transporter activity”, “vacuole”, “transmembrane transport”, Fig. 4c). This could indicate that that the turgor pressure build-up and H_2_O_2_ cytosol influx decreases from late buds to fruits only in shattering samples. In non-shattering samples these transcriptional changes were not detected, suggesting that aquaporins levels are stable and do not change drastically between stages.

Another area where we found evident trends from our DEG analysis is cell wall regulation. From the first to the last stage, there is a decline in the number of differentially expressed genes (DEGs) related to the cell wall: there is intensified cell wall modification at the first stage, conflicting signals by the late buds and flower stage, and a very small amount of DEGs associated with the cell wall on the fruit stage.

This suggests that the abscission layer forms early, with the main events happening in the early buds. This aligns with the results of the histological survey: in shattering samples the AZ is formed at early stages. Also, this observation is supported by the amounts of DEGs detected in simple phenotypic comparisons and complex stage-phenotype comparisons (Fig. 3). The main work on building the AZ appears to happen very early in flower development.

The double comparison between stages and phenotypes [shattering stage2 vs. stage1] – [nonshattering stage2 vs. stage1] suggests enhanced formation of the primary cell wall on the late buds stage1 comparing to stage2. This can seem contradictory from the first glance, but this just confirms the fact that early buds and late buds differ more in shattering plants. The enhanced cell wall biogenesis in late buds vs. early buds is indicated by increased biosynthesis of (1→3)-β-D-glucan—a component of the middle lamella—and the upregulation of pathways involved in primary cell wall formation, such as cellulose synthase, pectin biosynthetic processes, and protein retention in the Golgi apparatus. Polysaccharides of the cell wall matrix, including pectin and hemicelluloses, are formed solely within the Golgi cisternae and subsequently transported to the cell surface via Golgi-derived vesicles [62–64].

The flower and the fruit stages require a particular attention. At the fruit stage, based on previous studies, we anticipated the activation of genes involved in dissolving the pectin-rich middle lamella. Transcriptome analyses of tomato, soybean, and *A. thaliana* have identified a suite of cell wall-modifying enzymes active during the final stages of abscission, including cellulases (beta-1,4-endoglucanases, CELs), polygalacturonases, expansins and pectinesterases [56, 65]. However, in our study, only pectinesterase was differentially expressed on the fruit stage and, remarkably enough, it was downregulated. Also, on the fruit stage we observed the downregulation of laccase and beta-D-xylosidase. This implies altered cell wall dynamics but in a somewhat unexpected way, given previous findings. Notably, plants with shattering fruits also had shedding non-pollinated flowers. At the flower stage, extensin domain-containing protein and xyloglucan endotransglucosylase/hydrolase (XTH) genes were upregulated in shattering samples. XTH is the protein which is expected to be involved in abscission zone activation [66]. This suggests that activation of abscission layer activation begins earlier, not in fruits, as expected. The events related to the AZ activation on the fruit stage are evident in double comparison between stages and phenotypes [shattering stage4 vs. stage3] – [nonshattering stage4 vs. stage3], where the fruit stage show the upregulation of cellulase and glucan endo-1,3-beta-glucosidase, comparing to the flower stage (Table S14). We suggest the fruit stage may involve only the “final touch” in completing abscission zone activation.

Lastly, we note that in shattering samples at stage 2 (relative to stage 1), there is downregulation of genes related for cell cycle changes and cell division (e.g., DNA replication initiation, nuclear chromosome segregation, DNA polymerase processivity factor activity), pointing to reduced mitosis. Naturally, as the bud grows, the rate of cell division does decrease. However, in our experimental design, this reduction in cell division intensity in late-stage buds is specifically characteristic of shattering samples; in other words, in shattering samples, the intensity of cell division in later buds declines more than in non-shattering ones. In many studies the cells which build up the AZ are described as meristematic cells [5, 15]. The changes in life cycle status in shattering average pedicel cells could be associated with the meristematic nature of the cells composing AZ. Alternatively, it could result from the activity of a developmental gene, which triggers the establishment and differentiation of the separation layer. These two hypotheses are not contradictory—in fact, they may well complement one another.

### Candidate genes

Our current effort on the mapping of the shattering yielded a very wide region, spanning for the large part of chromosome 4. The main reason of this is the insufficient recombination frequency. The causative locus, based on the position of SNP frequency peak, is located near the middle of the chromosome **(**Fig. 6a-c**).** As in buckwheat the chromosomes are metacentric or submetacentric [67], this means that it is located near the centromere. And there is well a known phenomenon of lower recombination rate in the regions adjacent to centromeres [68]. The analysis of shattering and non-shattering individuals confirmed that the non-shattering plants either inherit the entire chromosome 4 from the non-shattering parent, or have a recombinant chromosome 4, with recombination occurring at the ends of the chromosome. The opposite situation is observed in the shattering samples: their chromosome 4 is either fully inherited from the shattering parent, or has recombination events at the ends **(**Fig. 6d**).** Another reason for low recombination rate might be the high divergence between parental accessions which belong to different subspecies (and this is the only possible combination of parents because only *F. esculentum* ssp. *ancestrale* is shattering). Thus the mapping of this particular trait using recombination-based methods might be challenging and should be complemented by alternative approaches, in particular, the consideration of candidate genes. Based on the studies on other dicots the genes encoding MADS-box family of transcription factors (TFs) are plausible candidates. Indeed, in tomato, two MADS-box TFs are known to directly regulate fruit abscission. One of these genes is homologous to the *A. thaliana* MADS-box flowering regulator SHORT VEGETATIVE PHASE (SVP), while the other is a homolog of the *A*. *thaliana* floral organ identity gene SEPALLATA4 (SEP4) [2]. In Arabidopsis, the genes SHATTERPROOF1 (SHP1) and SHATTERPROOF2 (SHP2) play critical roles in fruit dehiscence. These genes are redundant, only the double mutants (shp1 shp2) exhibit a non-dehiscent phenotype [44]. According to our results, chromosome 4 contains 15 MADS-box genes, four of which are expressed in the pedicels and are thus plausible candidates for the shattering locus. Among them there are the genes orthologous to ones with known function in abscission, in particular, tomato gene JOINTLESS, mentioned above (listed in the Table 2 under the name g13446.t2). Another tomato MADS-box, MACROCALYX, which also plays a role in abscission is an ortholog of APETALA1 of *A. thaliana*. Buckwheat genome has three co-orthologs of AP1, two are located on chromosomes 1 and third on chromosome 4 (listed in the Table 2 under the name g14228.t2). However the latter is unlikely to be a plausible candidate as individual graphs of allele frequencies show (Fig. S7) that several shattering plants have the variant of non-shattering parent in the region of localization of this gene. In some cases analysis of gene expression levels can help to prioritize candidate genes: indeed, the mutant phenotype might caused by the changes in expression (if a gene is lost or affected by transposon insertion or the mutation occurred in the regulatory region of a gene

-see, for example, [69]). However, none of the MADS-box genes located on chromosome 4 is differentially expressed between shattering and non-shattering plants and vice versa, DE genes located on this chromosome do not provide a plausible candidate.

## Conclusion

Abscission in common buckwheat is caused by the presence of an abscission zone consisting of 5–7 rows of small, tabular-like cells. Later in the development the upper (closer to the fruit) row of AZ cells lignifies. In non-shattering cultivar the AZ still can be detected, at least at some developmental stages but the level of its manifestation is much lower and more variable. It is not functional; in particular, it lacks lignification. Transcriptomic profiling done for shattering and non-shattering pedicels at four stages of development, have shown that most events occur at the transition from early to late bud. Among these events the most pronounced are stress response (presumably due to the action of abscisic acid) and the expression of enzymes involved in reconfiguration of the cell wall. Bulk segregant analysis has revealed that the causative locus is located at chromosome 4. More exact localization requires further study due to low recombination frequency at the associated region.

## Supplementary Information

Supplementary Material 1.

Supplementary Material 2.

## Data Availability

The datasets supporting the conclusions of this article are available in the ENA repository (https://www.ebi.ac.uk/ena/browser/home). Accessions: PRJEB86836, PRJEB86072, PRJEB87053 **.**The custom scripts used in this research are available on GitHub. Links to the scripts: [https://doi.org/10.5281/zenodo.15036165](https:/doi.org/10.5281/zenodo.15036165) (Recombination\_check), [https://doi.org/10.5281/zenodo.15036179](https:/doi.org/10.5281/zenodo.15036179) (Snp\_filtering). Full size images are available on Figshare: [https://doi.org/10.6084/m9.figshare.28603820](https:/doi.org/10.6084/m9.figshare.28603820).
