# Supplementary material for: Seed shattering in common buckwheat (Fagopyrum esculentum): insights from RNA-seq and morphological analysis
Source: BMC Plant Biol. 2025 Oct 14;25:1371. doi: 10.1186/s12870-025-07310-2 (PMC12522793; doi:10.1186/s12870-025-07310-2)
Supplement: Supplementary file 1 — Supplementary Material 1. [file 12870_2025_7310_MOESM1_ESM.docx]

**Supplementary Materials**

**Supplementary Figures**


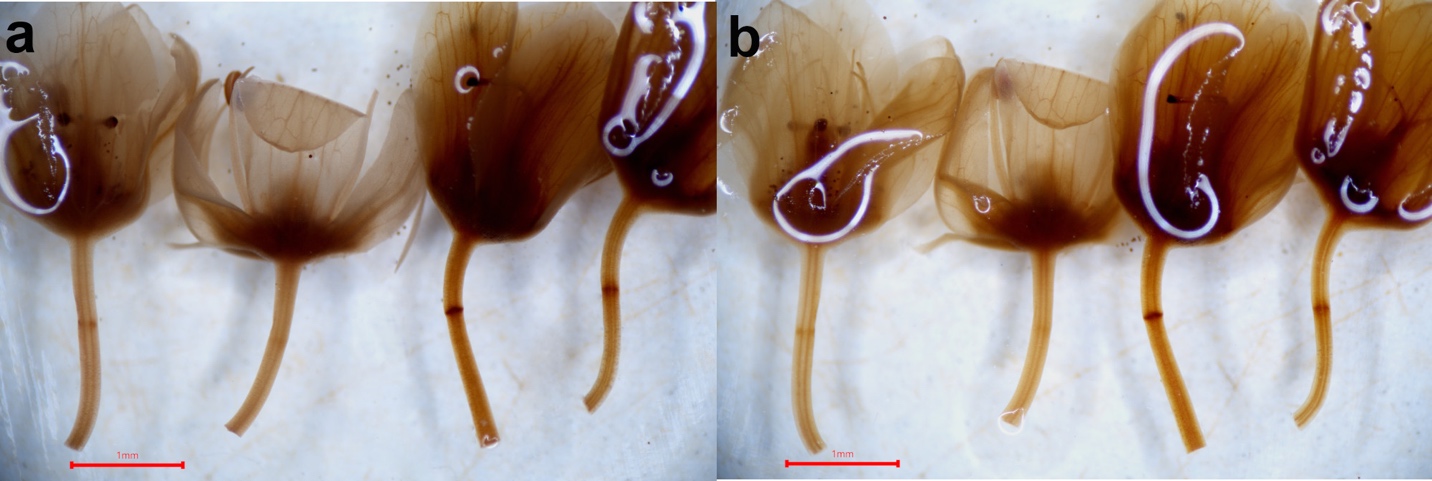


**Supplementary Fig. 1.** *Fagopyrum esculentum* cv. Dasha pedicel visualization in mature flowers; phloroglucinol reaction. Flowers from the same inflorescence demonstrate different expressivity of the AZ. Picture **a** is captured without staining, picture **b** demonstrate the result of phloroglucinol reaction.


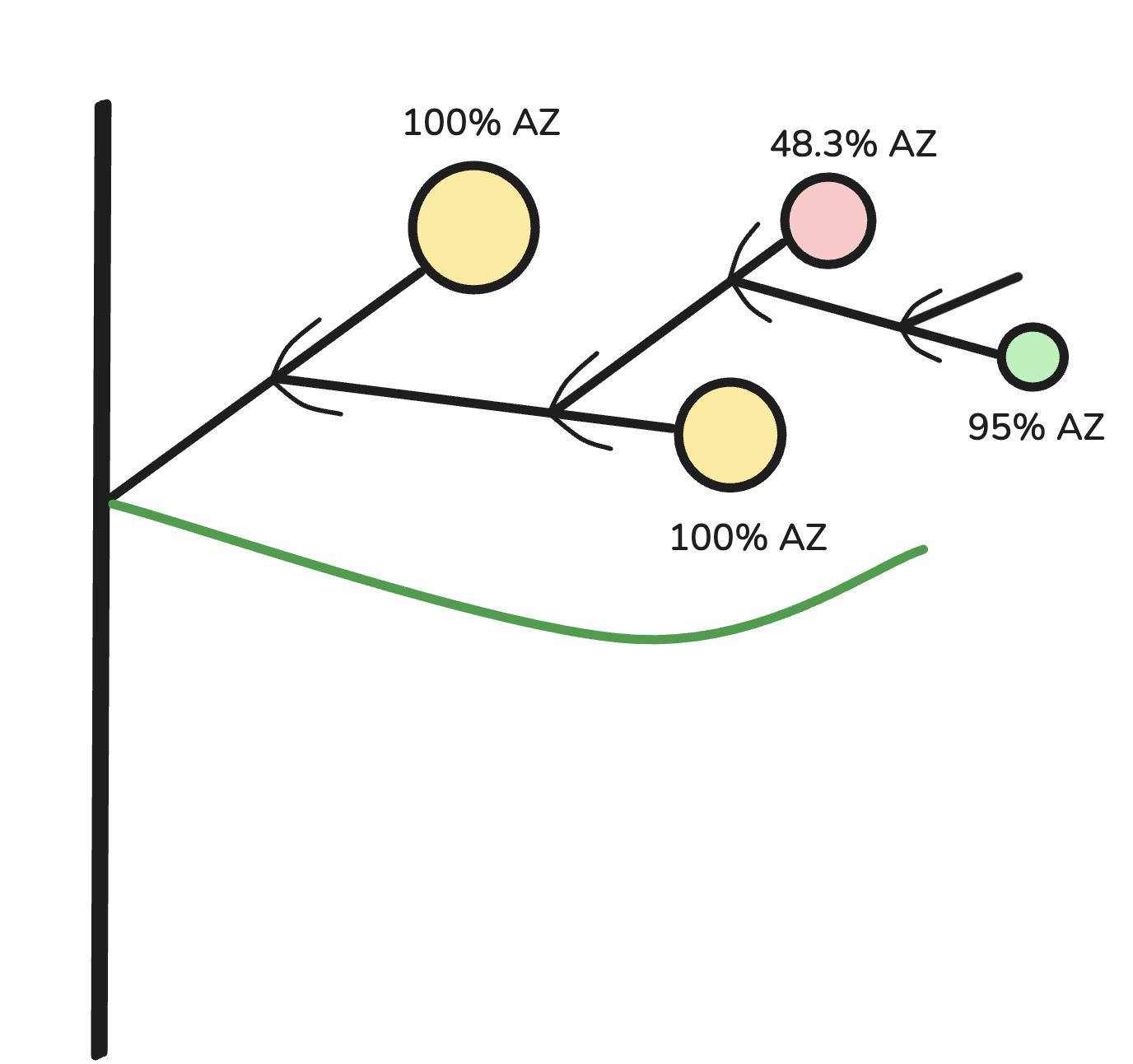


**Supplementary Fig. 2.** The proportion of *Fagopyrum esculentum* cv. Dasha flowers which have a clearly visible AZ, regarding their distribution within the elementary inflorescence. The youngest flower is the most distant from the inflorescence axis. We examined four oldest flowers in 60 elementary inflorescences. The flowers depicted with yellow color are senescent, they always had a visible AZ (56/56 flowers). The one which is highlighted with pink color is the anthetic flower, it has 48.3% of samples with visible AZ (29/60 flowers). The one highlighted with green is a flower bud, it has a visible AZ with 95% frequency (57/60 flowers). The smallest buds in the inflorescence were not analyzed.

**
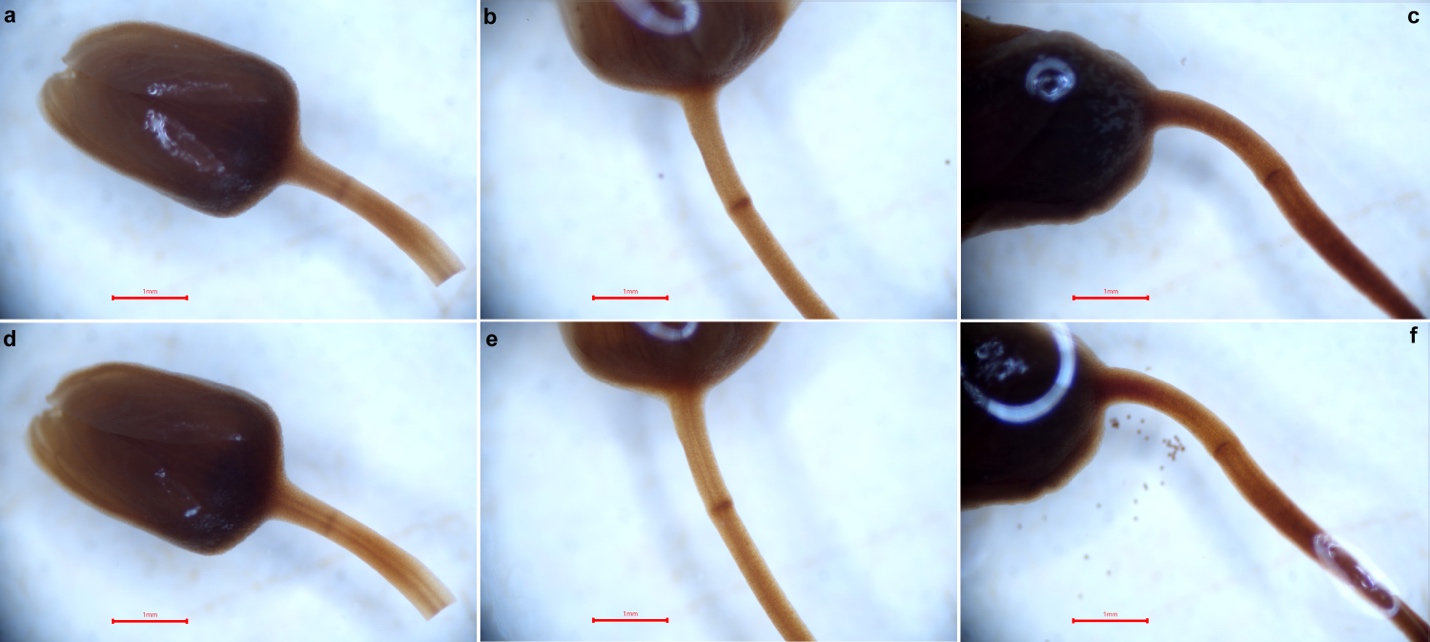
**

**Supplementary Fig 3.** *Fagopyrum esculentum* cv. Dasha lignin visualization: **a**, **d** in flower bud, **b**, **e** in mature flower, **c**, **f** in fruit. Pictures **a**, **b**, **c** are captured without staining, while pictures **d**, **e**, **f** demonstrate the result of phloroglucinol reaction.


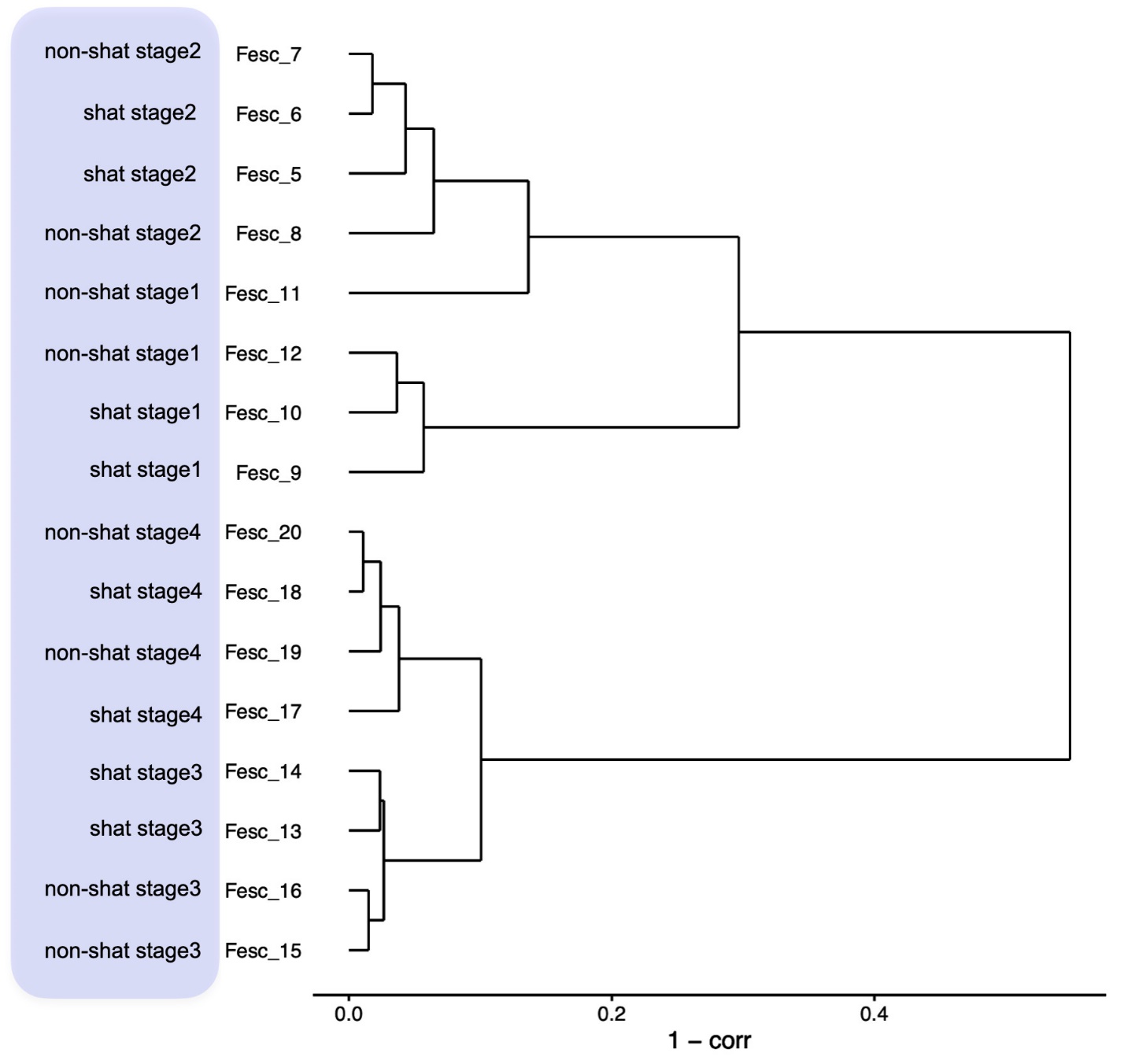


**Supplementary Fig. 4.** Hierarchical clustering dendrogram based on distance (1 - Pearson correlation) of gene expression profiles. Samples are mostly clustered by stage.


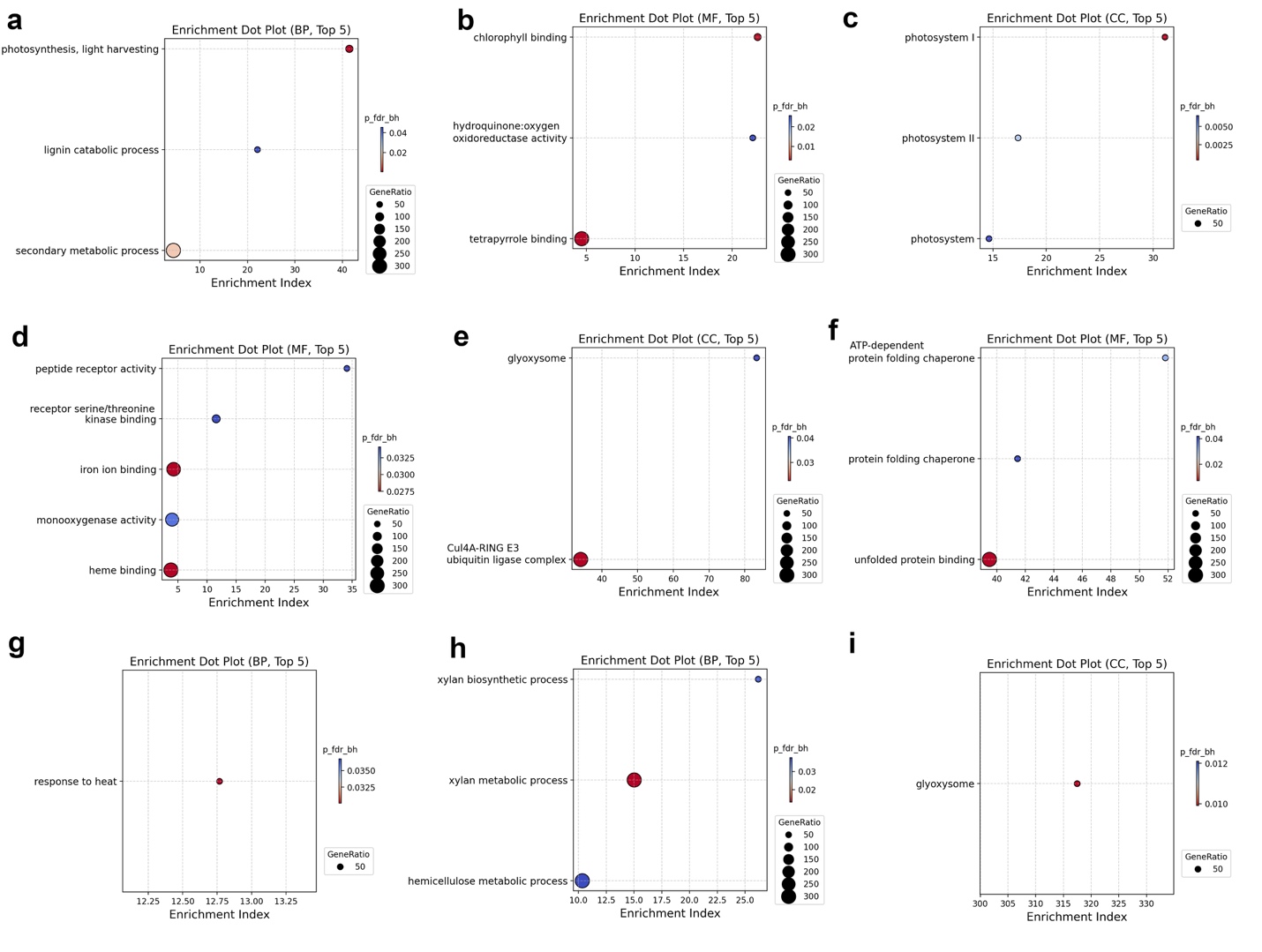


**Supplementary Fig. 5.** Enriched GO categories. Molecular function (MF) and Biological process (BP), Cellular Component (CC). **a-c** Upregulated genes stage1, shat vs. nonshat **d-e** Downregulated genes stage1, shat vs. nonshat **f-g** Upregulated genes stage2, shat vs. nonshat **h** Downregulated genes stage3, shat vs. nonshat **i** Down regulated genes stage4, shat vs. nonshat


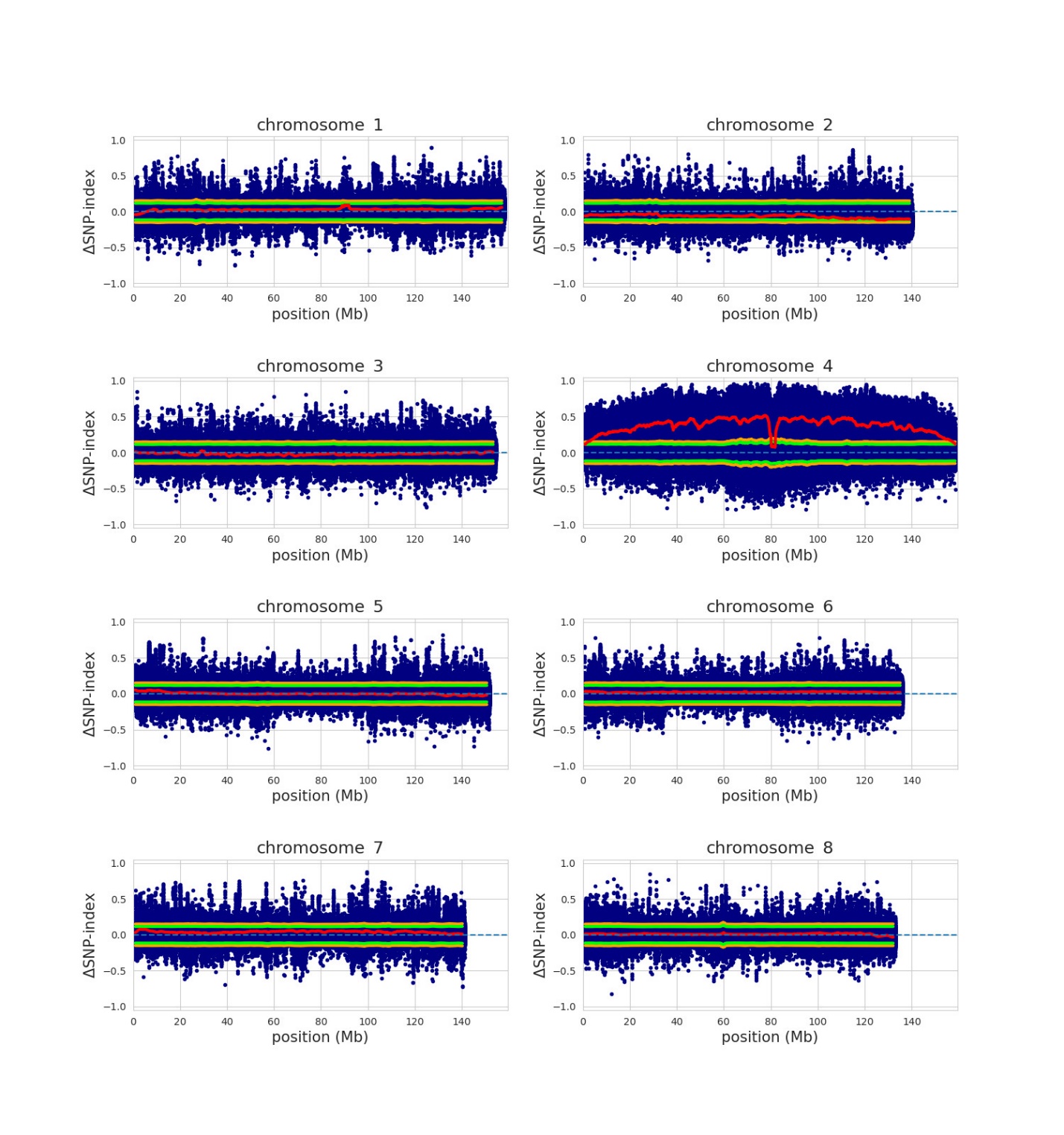


**
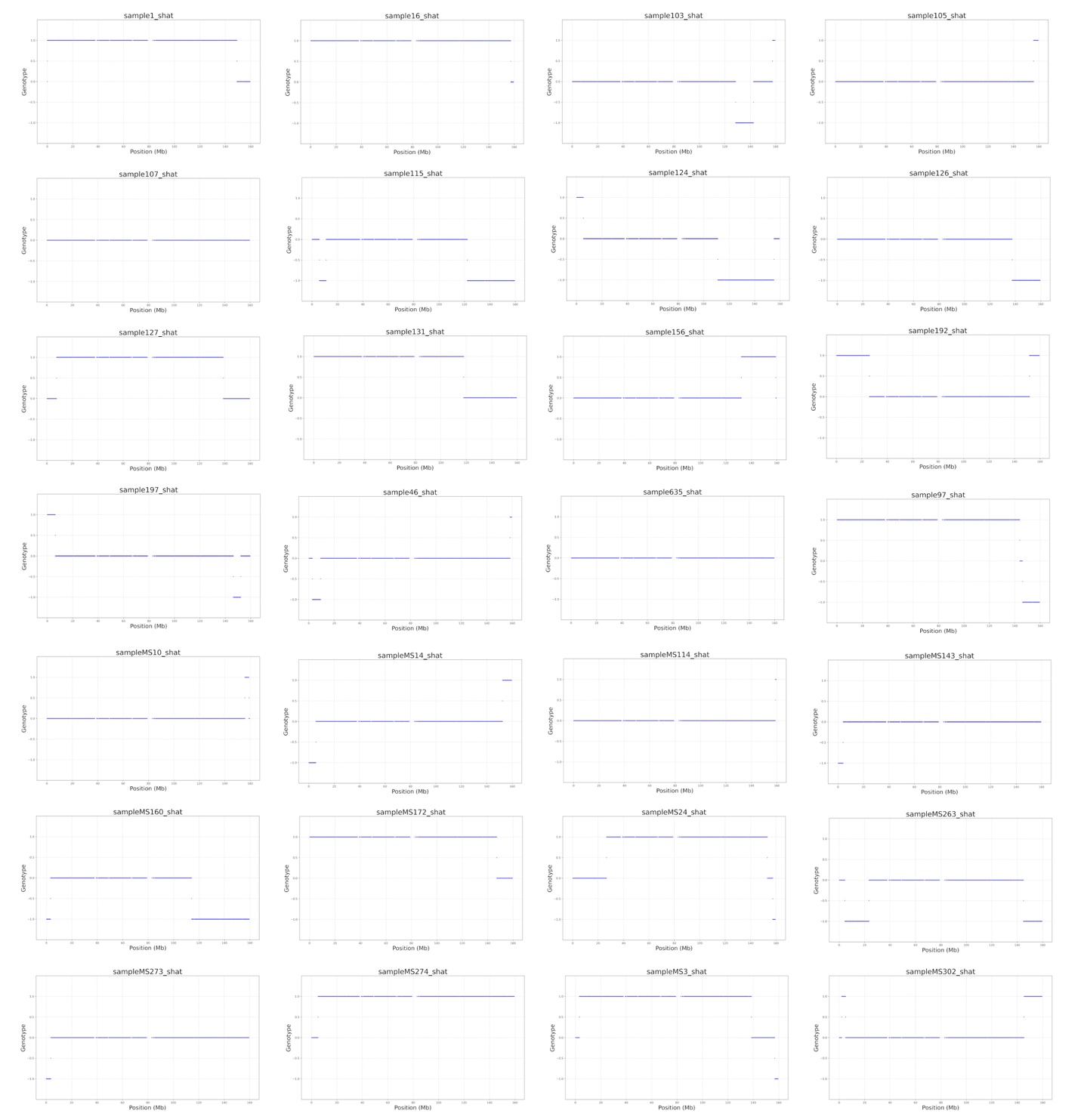
**

**Supplementary Fig. 6.** QTL-seq applied to *Fagopyrum esculentum* x *Fagopyrum esculentum* ssp. ancestrale F2 progeny identifies quantitative trait loci (QTLs) involved in seed shattering. Single nucleotide polymorphism (SNP)-index plots of ∆SNP-index plot with statistical confidence intervals under the null hypothesis of no QTLs (red, mean SNP-index; green, P < 0.05; yellow, P < 0.01). Basic filtration on mapping quality and coverage of SNP, number of SNPs = 35821013.

**Supplementary Fig. 7.** Genotype plot for individually sequenced F2 descendants (*Fagopyrum esculentum* ssp. *ancestrale* × *Fagopyrum esculentum* Dasha), focusing shattering samples, the fourth chromosome. The X-axis represents the position along the chromosome, and the Y-axis shows the genotype: «1» indicates homozygous alleles differing from the reference, «0» indicates heterozygous alleles, and «-1» indicates homozygous alleles identical to the reference. The reference genome is a non-shattering cultivar, and the observed genotype distribution aligns with expected inheritance patterns.
